# Microstructural White Matter Alterations in Pediatric Idiopathic Intracranial Hypertension: A Diffusion Tensor Imaging Study

**DOI:** 10.3390/children12121704

**Published:** 2025-12-17

**Authors:** Bilge Özgör, Hüseyin Ayvaz, Mahir Tan, Sevgi Demiröz Taşolar, Gül Yücel, Işınsu Bıçakcıoğlu, Serdal Güngör

**Affiliations:** 1 Division of Pediatric Neurology, Department of Pediatrics, Faculty of Medicine, İnönü University, Malatya 44050, Türkiye; mahir.tan@inonu.edu.tr (M.T.); gulyucel@inonu.edu.tr (G.Y.); isinsu.bicakcioglu@inonu.edu.tr (I.B.); 2 Department of Pediatric Radiology, Faculty of Medicine, İnönü University, Malatya 44050, Türkiye; huseyin.ayvaz@inonu.edu.tr (H.A.); sevgi.tasolar@inonu.edu.tr (S.D.T.); 3 Department of Pediatric Neurology, Antalya Medical Park Hospital, Antalya 07010, Türkiye; serdal.gungor@medicalpark.com.tr

**Keywords:** idiopathic intracranial hypertension, diffusion tensor imaging, fractional anisotropy, pediatric MRI, white matter

## Abstract

**Highlights:**

**What are the main findings?**

- Fractional anisotropy values in the optic radiation and posterior limb of the internal capsule were significantly reduced in pediatric idiopathic intracranial hypertension, indicating microstructural white matter alterations detectable by diffusion tensor imaging.
- DTI metrics—particularly FA—demonstrated strong discriminative accuracy (AUC = 0.83) for distinguishing affected patients from healthy controls, outperforming conventional MRI markers.

**What are the implications of the main findings?**

- DTI may serve as a complementary tool to conventional MRI in the diagnostic evaluation of pediatric IIH, providing quantitative insights into pressure-related white matter changes.
- These findings suggest that advanced diffusion imaging could aid early detection and monitoring of intracranial pressure–related alterations, warranting validation in larger, prospective pediatric cohorts.

**Abstract:**

**Background/Objectives**: Idiopathic intracranial hypertension (IIH) is an uncommon but clinically important cause of elevated intracranial pressure in children. Conventional MRI findings such as perioptic subarachnoid space (SAS) distension and posterior globe flattening are helpful but may lack sensitivity or specificity in certain cases. Diffusion tensor imaging (DTI), which quantifies white matter microstructure through metrics such as fractional anisotropy (FA), mean diffusivity (MD), axial diffusivity (AD), and radial diffusivity (RD), offers additional diagnostic potential, yet its role in pediatric IIH remains insufficiently defined. **Methods**: This retrospective case–control study included 26 pediatric patients with IIH and 26 age- and sex-matched controls who underwent brain MRI with DTI between 2010 and 2025. DTI parameters were measured in major white matter tracts, and conventional MRI findings associated with raised intracranial pressure were recorded. Associations between DTI metrics and conventional imaging markers were analyzed using standardized statistical tests. **Results**: Children with IIH demonstrated significantly reduced FA and increased MD and RD values in several key white matter regions, particularly within the optic radiation, splenium of the corpus callosum, and posterior limb of the internal capsule. FA values showed a negative correlation with perioptic SAS width, while RD and MD were positively correlated with posterior globe flattening and empty sella grade. Receiver operating characteristic analysis identified FA in the optic radiation as the strongest discriminator between IIH and controls (AUC = 0.83). Inter-observer reliability for FA measurements was excellent (ICC = 0.91). **Conclusions**: Pediatric IIH appears to be associated with pressure-related microstructural alterations in white matter, detectable through DTI. Among the diffusion metrics, FA demonstrated the strongest diagnostic potential and may serve as a complementary tool to conventional MRI. Validation in larger, prospective pediatric cohorts is required to establish its clinical utility.

## 1. Introduction

Idiopathic intracranial hypertension (IIH) is a clinical syndrome characterized by elevated cerebrospinal fluid (CSF) pressure in the absence of an underlying structural, metabolic, or infectious cause [1]. Although it predominantly affects young women, IIH can also occur in the pediatric population, where it represents a rare but clinically significant disorder. In children, diagnostic criteria are less clearly defined than in adults, and the interpretation of clinical and radiological findings, particularly in younger age groups, remains challenging. Because of the potential risk of permanent visual loss, early recognition and close monitoring are of paramount importance in this population [2,3,4]. In particular, pediatric IIH may present with greater clinical variability and less typical imaging patterns, further complicating early diagnosis [5].

The clinical presentation of pediatric IIH varies widely and commonly includes headache, papilledema, transient visual obscurations, diplopia, and nausea. Diagnosis is traditionally based on the modified Dandy criteria, which require elevated CSF opening pressure, normal CSF composition, and normal brain parenchyma without evidence of secondary causes. However, diagnostic uncertainty persists in children, especially in cases lacking papilledema or exhibiting atypical symptoms. In such settings, neuroimaging plays an essential role in supporting the diagnosis and excluding secondary etiologies [6,7,8].

Magnetic resonance imaging (MRI) serves as the cornerstone of IIH evaluation. Several radiological signs have been proposed as surrogate indicators of raised intracranial pressure, including perioptic subarachnoid space (SAS) distension, posterior globe flattening, empty sella, and transverse sinus stenosis. These findings have been consistently associated with IIH both in adults and children [9,10]. Among them, perioptic SAS enlargement demonstrates the highest sensitivity, while posterior globe flattening and transverse sinus stenosis provide high specificity for the diagnosis. In pediatric cohorts, an optic nerve sheath diameter greater than approximately 5.3–5.4 mm has been reported as a useful threshold for IIH detection [11,12,13]. Nevertheless, the diagnostic value of these conventional MRI markers remains limited, and there is a growing need for objective, quantitative imaging biomarkers that could enhance diagnostic accuracy.

Diffusion tensor imaging (DTI) is an advanced MRI technique capable of assessing white matter microstructure by quantifying the directional movement of water molecules. DTI provides several scalar metrics—fractional anisotropy (FA), mean diffusivity (MD), axial diffusivity (AD), and radial diffusivity (RD)—each reflecting distinct biological properties of neural tissue. FA is primarily associated with axonal organization, AD with axonal integrity, RD with myelin status, and MD with overall diffusivity and extracellular water content [14,15]. These parameters can detect subtle microstructural alterations long before they appear on conventional MRI, making DTI particularly valuable in pediatric neuroimaging [16,17].

In adult IIH studies, decreased FA and increased RD values have been reported in the optic nerves and optic radiations, suggesting that elevated intracranial pressure may induce microstructural disorganization [2,7,18]. Similar patterns of FA reduction and RD/ADC elevation have also been documented in spontaneous intracranial hypotension, implying that DTI metrics are sensitive to pressure-related changes across the cranial pressure spectrum [19,20]. Beyond IIH, DTI has been widely used in pediatric neurology to evaluate conditions such as optic pathway gliomas, demyelinating disorders, and traumatic brain injury, further demonstrating its value as an early marker of white matter alteration [21,22].

Despite these findings, the role of DTI in pediatric IIH has not yet been systematically investigated. Understanding whether elevated CSF pressure leads to measurable white matter changes in children could provide novel diagnostic and pathophysiological insights.

The present study aimed to evaluate the microstructural integrity of white matter tracts in children with idiopathic intracranial hypertension using diffusion tensor imaging. Specifically, we sought to analyze FA, MD, AD, and RD parameters in key white matter pathways and to explore their relationship with conventional MRI markers such as perioptic SAS distension, posterior globe flattening, empty sella, and transverse sinus stenosis, in order to determine the potential diagnostic value of DTI-derived metrics in pediatric IIH.

## 2. Materials and Methods

### 2.1. Study Design and Participants

This retrospective case–control study was conducted at the Faculty of Medicine, İnönü University (Malatya, Türkiye). Pediatric patients aged between 5 and 18 years who were diagnosed with idiopathic intracranial hypertension (IIH) and underwent brain magnetic resonance imaging (MRI) including a diffusion tensor imaging (DTI) sequence between 1 January 2010, and 31 December 2024, at the Department of Pediatric Neurology were included in the study.

The diagnosis of IIH was established according to the modified Dandy criteria, requiring age-adjusted elevated cerebrospinal fluid (CSF) opening pressure, normal CSF composition, and the absence of intracranial mass, infection, metabolic, or structural causes. The control group consisted of age- and sex-matched children who underwent MRI with DTI during the same period for other clinical indications (e.g., headache or seizure) but had normal imaging findings and no clinical or radiological evidence suggestive of IIH.

Demographic and clinical data, including presenting symptoms and ophthalmological findings, were obtained from the institutional electronic medical record system. Exclusion criteria included secondary intracranial hypertension, intracranial mass, hydrocephalus, infection, metabolic or mitochondrial disease, significant motion artifacts, major structural brain anomalies, or insufficient image quality that prevented accurate optic nerve evaluation.

A total of 52 participants—26 patients with IIH and 26 healthy controls—met the inclusion criteria and were included in the final analysis. The study was approved by the İnönü University Health Sciences Scientific Research Ethics Committee (Approval Date: 30 September 2025; Decision No: 2025/8476) and was conducted in accordance with the principles of the Declaration of Helsinki.

Children younger than 5 years were excluded to avoid diffusion variability related to incomplete myelination.

### 2.2. Imaging Protocol

All MRI examinations were performed using a 1.5 Tesla scanner (Siemens, Erlangen, Germany). The standard imaging protocol included axial, sagittal, and coronal T1-weighted, T2-weighted, and FLAIR sequences, as well as fat-suppressed and venographic (MRV) sequences when available.

Diffusion tensor imaging was performed using a single-shot echo-planar imaging (EPI) sequence with two b-values (b = 0 and b = 1000 s/mm^2^) and 30 noncollinear gradient directions. Acquisition parameters were as follows: repetition time (TR) = 4000 ms, echo time (TE) = 77 ms, field of view (FOV) = 220 mm, matrix size = 128 × 128, and slice thickness = 5 mm [23].

### 2.3. Diffusion Tensor Imaging Analysis

DTI datasets were processed using Siemens SyngoVia software (Version 2.0). Fractional anisotropy (FA), mean diffusivity (MD), axial diffusivity (AD), and radial diffusivity (RD) maps were automatically generated.

Regions of interest (ROIs) were manually delineated on color-coded FA maps, maintaining anatomical accuracy. Measurements were performed in the optic nerve (when technically feasible), optic radiation, splenium of the corpus callosum, corticospinal tract, and the anterior and posterior limbs of the internal capsule (ALIC and PLIC). Each ROI covered an area of approximately 20–30 mm^2^.

All ROIs were placed bilaterally, and left–right measurements were averaged for analysis to enhance robustness.

No mirroring tool was used; each ROI was manually drawn on both sides to avoid interpolation-related artifacts and to ensure anatomical precision.

All measurements were independently performed by two blinded raters (a pediatric neurologist and a radiologist). When discrepancies were noted, the mean of the two measurements was used for analysis. Inter-observer agreement was evaluated using the intraclass correlation coefficient (ICC).

Conventional MRI parameters, including perioptic subarachnoid space (SAS) distension, posterior globe flattening, empty sella, and transverse sinus stenosis, were also assessed. The optic nerve sheath diameter was measured 3 mm posterior to the globe, perpendicular to the long axis of the optic nerve. Posterior globe flattening was defined as loss or concavity of the posterior scleral contour [24].

**Figure 1:**
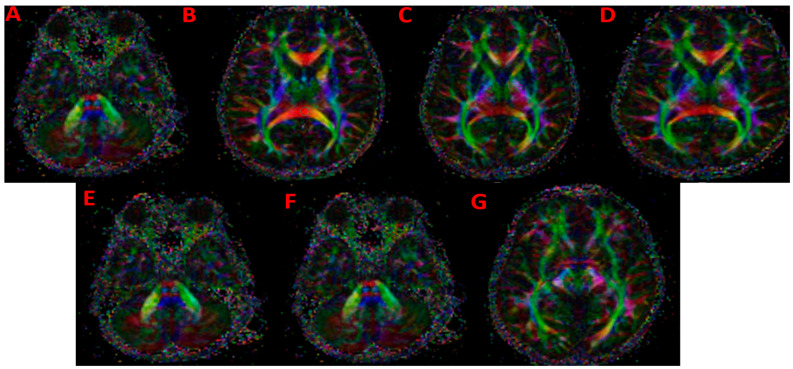
Directionally color-coded fractional anisotropy (FA) maps demonstrating the representative placement of all manually drawn regions of interest (ROIs) used for diffusion tensor imaging (DTI) analysis. (**A**) ROI placed over the left optic radiation, capturing the lateral geniculostriate fibers; (**B**) ROI placed on the splenium of the corpus callosum, representing interhemispheric commissural fibers; (**C**) ROI placed on the left posterior limb of the internal capsule (PLIC), covering the posterior thalamocortical projection fibers; (**D**) ROI placed on the left anterior limb of the internal capsule (ALIC), including anterior thalamic and frontopontine tracts; (**E**) ROI placed on the left corticospinal tract at the level of the cerebral peduncle; (**F**) ROI placed on the left optic nerve, manually delineated on the proximal intraorbital segment; (**G**) ROI placed on the right optic radiation as an additional illustration of tract-level sampling. All ROIs were positioned on color-coded FA maps to ensure anatomical accuracy and reproducibility across participants.

Figure 1 illustrates the representative placement of all manually drawn regions of interest (ROIs), including the optic radiation, splenium of the corpus callosum, anterior and posterior limbs of the internal capsule, corticospinal tract, and optic nerve.

### 2.4. Statistical Analysis

All statistical analyses were performed using IBM SPSS Statistics version 28.0 (IBM Corp., Armonk, NY, USA). Continuous variables were tested for normality using the Shapiro–Wilk test. Normally distributed data were expressed as mean ± standard deviation (SD), and non-normally distributed data as median and interquartile range (IQR). Categorical variables were presented as frequencies and percentages.

Between-group comparisons of continuous variables were performed using the independent samples Student’s *t*-test for normally distributed data and the Mann–Whitney U test for nonparametric variables. The Chi-square or Fisher’s exact test was used to compare categorical variables when appropriate. In accordance with SAMPL guidelines, each *p*-value presented in the tables is explicitly accompanied by the corresponding statistical test used to generate that value.

The associations between diffusion tensor imaging (DTI) parameters and conventional MRI findings were evaluated using Pearson’s correlation coefficient for normally distributed data and Spearman’s rank correlation coefficient for non-normally distributed data. Correlation strength was interpreted as weak (r = 0.20–0.39), moderate (r = 0.40–0.59), or strong (r ≥ 0.60). Correlation *p*-values are likewise reported together with the corresponding correlation test.

Receiver operating characteristic (ROC) curve analysis was used to assess the diagnostic performance of DTI parameters. AUC values with 95% confidence intervals were calculated, and optimal cut-off points were determined according to the Youden index (J = sensitivity + specificity − 1). To avoid model instability associated with low event-per-variable (EPV) ratios in small datasets, multivariate logistic regression, machine-learning classifiers, and internal validation procedures (including bootstrapping, leave-one-out cross-validation, or training–test splitting) were not performed. Accordingly, ROC results represent unvalidated in-sample performance and should be interpreted with appropriate caution.

Inter-observer reliability for ROI-based FA measurements was evaluated using the two-way random-effects intraclass correlation coefficient (ICC). Agreement was categorized as poor (<0.50), moderate (0.50–0.74), good (0.75–0.89), or excellent (≥0.90).

Sample size estimation was performed using G*Power version 3.1.9.7 (Heinrich Heine University, Düsseldorf, Germany). Based on previously published adult idiopathic intracranial hypertension data showing an optic nerve FA AUC of 0.83 (Cohen’s d ≈ 1.34), a two-tailed α = 0.05 and power (1 − β) = 0.80 indicated a minimum of 10 participants per group for large effects. For smaller expected tract-level differences (d = 0.50–0.60), the required total sample size was 45 participants. The inclusion of 52 subjects (26 IIH and 26 controls) was therefore sufficient to achieve adequate statistical power for all analyses.

### 2.5. Classification and Validation Strategy

Univariate classification performance was evaluated using receiver operating characteristic curve analysis for each diffusion tensor imaging parameter. AUC values and optimal cut-off points were derived using the Youden index. No multivariate classification models (e.g., logistic regression or discriminant analysis) were constructed, as such analyses were beyond the scope of the present study.

Because of the modest sample size (*n* = 52), no training–test split, cross-validation, bootstrap resampling, or other internal validation procedures were applied. As a result, ROC values represent unvalidated, in-sample diagnostic performance and should be interpreted with caution. This methodological constraint has been addressed in the Limitations section.

Furthermore, internal validation methods such as bootstrapping or leave-one-out cross-validation were intentionally avoided because the low event-per-variable (EPV) ratio would lead to highly unstable and misleading classification estimates in small datasets. Therefore, univariate ROC analyses were reported without resampling-based validation to maintain methodological robustness.

## 3. Results

A total of 52 children were included in the analysis, consisting of 26 patients with idiopathic intracranial hypertension (IIH) and 26 age- and sex-matched healthy controls. As shown in Table 1, there was no significant difference between groups in terms of age (12.4 ± 3.1 vs. 12.1 ± 3.0 years, *p* = 0.72) or sex distribution (73.1% vs. 69.2%, *p* = 0.77). The mean body mass index (BMI) was significantly higher in the IIH group (23.1 ± 2.8 vs. 20.8 ± 2.5 kg/m^2^, *p* = 0.01). The most common clinical symptoms among patients with IIH were headache (84.6%), papilledema (65.4%), and visual disturbance (57.7%). All *p*-values are reported together with the corresponding statistical test, in accordance with SAMPL guidelines.

Radiological markers of elevated intracranial pressure demonstrated clear differences between groups. As presented in Table 2, perioptic subarachnoid space (SAS) dilation, posterior globe flattening, empty sella, and transverse sinus stenosis were all significantly more frequent among IIH patients (all *p* < 0.001).

Diffusion tensor imaging (DTI) analysis revealed significant microstructural alterations across several white matter tracts in the IIH group. As detailed in Table 3, fractional anisotropy (FA) values were significantly lower in the optic radiation, splenium of the corpus callosum, and posterior limb of the internal capsule (PLIC), while mean diffusivity (MD) and radial diffusivity (RD) were significantly higher in these regions (all *p* ≤ 0.04). Axial diffusivity (AD) demonstrated moderate increases in multiple tracts.

Significant correlations were identified between DTI measures and conventional MRI findings. As shown in Table 4, perioptic SAS width demonstrated a strong negative correlation with FA in both the optic radiation (r = −0.56, *p* = 0.003) and the PLIC (r = −0.49, *p* = 0.009). Posterior globe flattening correlated positively with RD in the corpus callosum (r = +0.52, *p* = 0.006), while empty sella grade showed a positive correlation with MD in the splenium (r = +0.38, *p* = 0.04).

Receiver operating characteristic analysis demonstrated that FA in the optic radiation had the highest discriminative performance, with an AUC of 0.83 (95% CI, 0.73–0.93; *p* < 0.001). As detailed in Table 5, FA in the PLIC and RD in the corpus callosum also showed good diagnostic accuracy, while MD in the corticospinal tract yielded moderate predictive value. Inter-observer agreement for FA measurements was excellent (ICC = 0.91; 95% CI, 0.84–0.95; *p* < 0.001). These ROC values represent unvalidated in-sample diagnostic performance, as no internal validation was applied.

**Figure 2:**
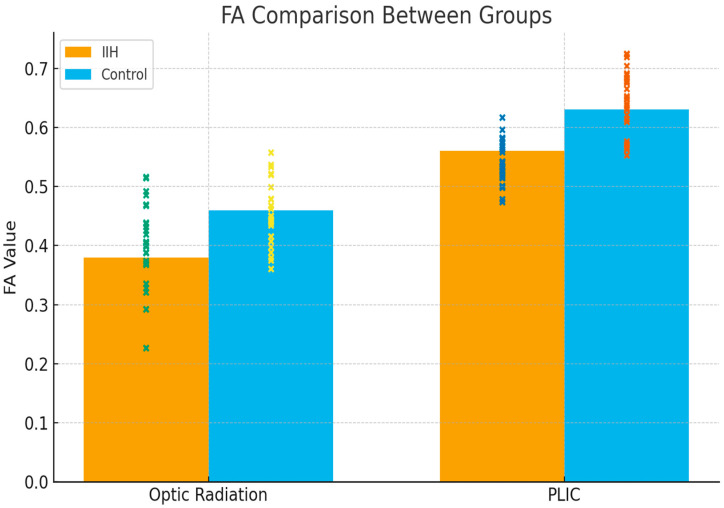
Comparison of mean fractional anisotropy (FA) values between idiopathic intracranial hypertension (IIH) patients and healthy controls in key white matter tracts, including the optic radiation, splenium of the corpus callosum, anterior limb (ALIC) and posterior limb (PLIC) of the internal capsule, and corticospinal tract. Error bars represent standard deviations.

As illustrated in Figure 2, fractional anisotropy values in the optic radiation and PLIC were markedly reduced in patients with IIH compared with healthy controls, consistent with the tract-level differences summarized in Table 3.

Figure 2 Bar Plot of Fractional Anisotropy (FA) Values in Major White Matter Tracts.

**Figure 3:**
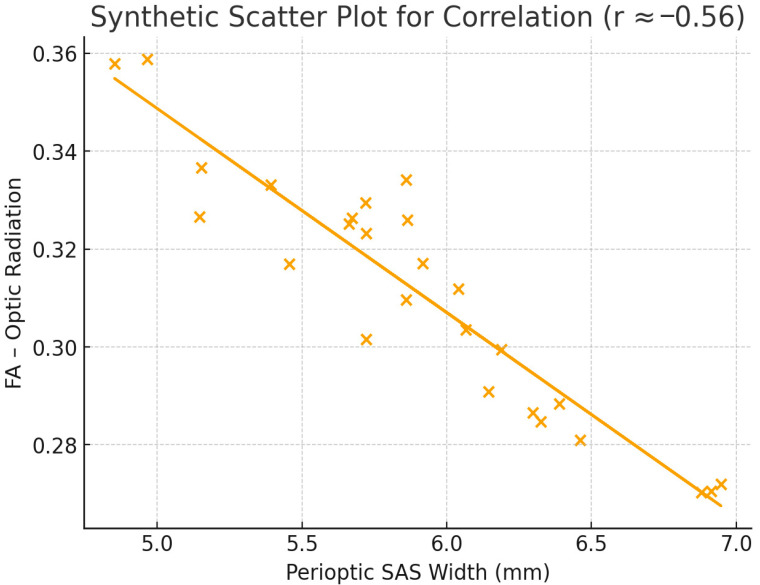
Scatter plot depicting the correlation between perioptic subarachnoid space (SAS) width and fractional anisotropy (FA) values in the optic radiation among patients with idiopathic intracranial hypertension. A significant negative correlation was observed (r = −0.56, *p* = 0.003), indicating that increased SAS distension corresponds to decreased microstructural integrity.

As shown in Figure 3, perioptic SAS width demonstrated a strong negative correlation with FA in the optic radiation (r = −0.56), supporting the association between raised intracranial pressure and microstructural white matter alteration.

Figure 3 Scatter Plot Showing Correlation Between Perioptic SAS Width and Optic Radiation FA.

## 4. Discussion

In this retrospective case–control study, diffusion tensor imaging (DTI) parameters were evaluated in pediatric patients with idiopathic intracranial hypertension (IIH) to investigate possible microstructural alterations in white matter tracts. Our findings revealed significantly lower fractional anisotropy (FA) and higher mean diffusivity (MD) and radial diffusivity (RD) values in children with IIH compared with healthy controls, suggesting that chronically elevated intracranial pressure may lead to subtle but measurable microstructural disruptions in the developing brain. These changes are biologically plausible given that FA reductions commonly reflect axonal disorganization, whereas increases in RD typically indicate myelin sheath alterations and impaired white matter coherence.

Previous adult studies have reported decreased FA and increased RD values, particularly within the optic nerve and optic radiation, in patients with IIH [25,26]. These alterations have been interpreted as evidence of impaired axonal alignment and myelin disorganization secondary to elevated cerebrospinal fluid (CSF) pressure. Our results align with these findings, demonstrating reduced FA and increased RD in the optic radiation and posterior limb of the internal capsule (PLIC). The strong negative correlations between FA values and perioptic subarachnoid space (SAS) width further support the notion that mechanical expansion of CSF spaces may directly influence adjacent white matter microstructure through compression, venous congestion, or altered interstitial fluid dynamics [27,28].

Beyond the optic pathway, our results indicate that pressure-related microstructural changes may be more widespread. Alterations within the splenium of the corpus callosum and corticospinal tract suggest that idiopathic intracranial hypertension may exert a global impact on white matter integrity. Similar observations have been made in adult cohorts using tract-based spatial statistics, where elevated intracranial pressure was associated with reduced FA in multiple supratentorial pathways, including the corpus callosum and thalamocortical tracts [29,30]. Such diffuse involvement may reflect a combination of mechanical strain, impaired glymphatic flow, microvascular dysfunction, and impaired axonal–glial coupling rather than isolated demyelination.

Posterior globe flattening and empty sella are well-established imaging indicators of elevated intracranial pressure [9,10,31]. Also showed meaningful associations with diffusion metrics in our cohort. The positive correlation between posterior globe flattening severity and RD in the corpus callosum suggests that macroscopic deformation of the posterior sclera may mirror microstructural disorganization in connected visual pathways. Similarly, the association between empty sella grade and increased MD in the splenium may represent pressure-related extracellular water expansion or microcirculatory disruption. These multimodal parallels reinforce the growing view that IIH produces both macroscopic and microscopic injury patterns detectable with complementary neuroimaging techniques [32,33].

Receiver operating characteristic (ROC) analysis demonstrated that FA in the optic radiation had the highest discriminatory performance (AUC = 0.83). This finding is consistent with adult studies in which the optic radiation appears highly sensitive to intracranial pressure fluctuations [26,34]. Given that FA reflects directional coherence of axonal fibers, its marked reduction in IIH may be attributable to pressure-induced axonal stretch, mechanical compression, or myelin disruption. However, the diagnostic applicability of these metrics remains preliminary. Variability in acquisition parameters, developmental influences on diffusion values, and operator-dependent ROI selection represent important practical considerations in clinical translation [35].

To our knowledge, this study represents one of the first systematic DTI analyses performed in pediatric IIH. Because myelination and axonal maturation progress substantially throughout childhood and adolescence, microstructural responses to intracranial pressure may differ from adults. Developmental studies have shown age-related increases in FA and decreases in RD, particularly in posterior white matter tracts [36]. Therefore, pressure-induced deviations from expected developmental trajectories may be particularly relevant in pediatric populations, reinforcing the potential value of DTI in this setting.

Our findings also align with broader pediatric neurology research in which DTI has been used to detect early microstructural alterations in conditions such as traumatic brain injury, optic pathway gliomas, demyelinating disease, and mitochondrial disorders. These examples underscore that DTI is a sensitive modality for capturing microstructural changes even when conventional MRI appears normal, supporting its potential value in pediatric IIH.

This study has several strengths. The patient cohort was homogeneous, imaging protocols were standardized, and inter-observer agreement for ROI-based FA measurements was excellent (ICC = 0.91), enhancing measurement reliability. The integration of conventional MRI findings with quantitative DTI metrics provides a multimodal perspective on the structural consequences of elevated intracranial pressure.

However, several limitations must be acknowledged. First, the retrospective design introduces a potential risk of selection bias, and the modest sample size limits the ability to perform meaningful subgroup analyses. Second, the ROI-based approach, although practical in pediatric imaging, is operator-dependent and inherently less sensitive than voxelwise techniques such as tract-based spatial statistics (TBSS). Third, diffusion tensor imaging is susceptible to motion and susceptibility artifacts, which are particularly common in pediatric populations and may influence measurements despite standardized acquisition procedures. Fourth, quantitative cerebrospinal fluid pressure values were not available for all patients, limiting the ability to establish direct physiological correlations. Fifth, visual function outcomes and optic disc morphology were not assessed, which reduces the clinical interpretability of the imaging findings. Sixth, the archived DTI scans used for Figure 1 had limited spatial resolution, constraining the clarity with which ROI placement could be visually demonstrated. Seventh, and most importantly, no validation procedures—such as cross-validation, bootstrapping, or train–test splitting—were applied to the ROC analyses due to the relatively small sample size. Consequently, the reported diagnostic accuracy values should be regarded as exploratory and may overestimate true performance. Finally, the cross-sectional design precludes conclusions regarding causality or the potential reversibility of the observed diffusion abnormalities.

## 5. Conclusions

In conclusion, this study demonstrates that idiopathic intracranial hypertension in children is associated with detectable alterations in white matter microstructure, as evidenced by reduced fractional anisotropy and increased diffusivity metrics in multiple neural pathways. The optic radiation and posterior limb of the internal capsule appear particularly susceptible to pressure-related disruption, consistent with their anatomical proximity to CSF compartments and their developmental vulnerability. These findings suggest that DTI may provide complementary quantitative insights beyond conventional MRI and may hold promise as an adjunctive biomarker in the evaluation of pediatric IIH. However, given developmental variability, methodological constraints, and the lack of validation analyses, these results should be interpreted with caution. Future multicenter prospective studies with larger cohorts, longitudinal follow-up, and advanced diffusion modeling (e.g., NODDI, DMI) are essential to confirm the diagnostic and prognostic utility of DTI in this vulnerable population.

## Figures and Tables

**Table 1:** Demographic and clinical characteristics of the study population.

Parameter	IIH (*n* = 26)	Control (*n* = 26)	*p*-Value
Age (years, mean ± SD)	12.4 ± 3.1	12.1 ± 3.0	0.72
Female sex, n (%)	19 (73.1)	18 (69.2)	0.77
BMI (kg/m^2^, mean ± SD)	23.1 ± 2.8	20.8 ± 2.5	0.01
Headache, n (%)	22 (84.6)	–	–
Visual disturbance, n (%)	15 (57.7)	–	–
Papilledema, n (%)	17 (65.4)	–	–

Statistical tests: Independent samples *t*-test (age, BMI), chi-square test (sex). Abbreviations: BMI, body mass index; SD, standard deviation.

**Table 2:** Conventional MRI findings in IIH and control groups.

MRI Finding	IIH (*n* = 26)	Control (*n* = 26)	*p*-Value
Perioptic SAS dilation	22 (84.6%)	1 (3.8%)	<0.001
Posterior globe flattening	18 (69.2%)	0 (0%)	<0.001
Empty sella (grade 3–4)	14 (53.8%)	1 (3.8%)	<0.001
Transverse sinus stenosis	16 (61.5%)	0 (0%)	<0.001
Optic nerve tortuosity	10 (38.5%)	2 (7.7%)	0.01

Statistical test: Chi-square test.

**Table 3:** Comparison of DTI parameters between IIH and control groups.

ROI	FA (Mean ± SD)	*p*	MD (×10^−3^ mm^2^/s)	*p*	AD	*p*	RD	*p*
Optic radiation	0.38 ± 0.06	<0.001	0.92 ± 0.08	0.002	1.22 ± 0.11	0.03	0.61 ± 0.09	<0.001
Splenium	0.64 ± 0.07	0.004	0.89 ± 0.06	0.04	1.21 ± 0.10	0.08	0.55 ± 0.08	0.02
PLIC	0.56 ± 0.05	<0.001	0.91 ± 0.07	0.001	1.18 ± 0.10	0.02	0.58 ± 0.07	<0.001
ALIC	0.54 ± 0.06	0.01	0.87 ± 0.09	0.06	1.16 ± 0.12	0.14	0.57 ± 0.10	0.04
Corticospinal tract	0.48 ± 0.07	0.02	0.94 ± 0.07	0.05	1.25 ± 0.09	0.08	0.63 ± 0.08	0.03
Optic nerve (subset)	0.31 ± 0.05	0.002	0.96 ± 0.09	0.01	1.27 ± 0.12	0.07	0.65 ± 0.09	0.004

Statistical tests: Independent samples *t*-test (DTI parameters); Mann–Whitney U test where appropriate. Abbreviations: FA, fractional anisotropy; MD, mean diffusivity; AD, axial diffusivity; RD, radial diffusivity.

**Table 4:** Correlation of DTI parameters with conventional MRI findings.

MRI Finding	DTI Metric	ROI	r	*p*-Value
Perioptic SAS width	FA	Optic radiation	−0.56	0.003
Perioptic SAS width	FA	PLIC	−0.49	0.009
Posterior globe flattening	RD	Corpus callosum	+0.52	0.006
Empty sella grade	MD	Splenium	+0.38	0.04
Transverse sinus stenosis	FA	Corticospinal tract	−0.35	0.06

Statistical test: Pearson correlation.

**Table 5:** Diagnostic performance of DTI metrics for differentiating IIH from controls.

DTI Metric	ROI	AUC (95% CI)	Cut-Off	Sensitivity	Specificity	*p*-Value
FA	Optic radiation	0.83 (0.73–0.93)	≤0.40	81%	77%	<0.001
FA	PLIC	0.79 (0.67–0.91)	≤0.54	76%	73%	0.002
RD	Corpus callosum	0.75 (0.61–0.88)	≥0.58	73%	69%	0.01
MD	Corticospinal tract	0.72 (0.58–0.85)	≥0.91	68%	71%	0.03
AD	ALIC	0.69 (0.54–0.84)	≤1.17	62%	66%	0.07

Statistical test: Cut-off values were determined using the Youden index, and confidence intervals for AUC values were calculated using DeLong’s method. Abbreviations: AUC, area under the curve; CI, confidence interval; FA, fractional anisotropy; MD, mean diffusivity; AD, axial diffusivity; RD, radial diffusivity; ROI, region of interest; PLIC, posterior limb of internal capsule; ALIC, anterior limb of internal capsule.

## Data Availability

The data presented in this study are available on reasonable request from the corresponding author. The data are not publicly available due to ethical and privacy restrictions.

## Abbreviations

AD	Axial diffusivity
ALIC	Anterior limb of internal capsule
AUC	Area under the curve
BMI	Body mass index
CI	Confidence interval
CSF	Cerebrospinal fluid
DTI	Diffusion tensor imaging
FA	Fractional anisotropy
FOV	Field of view
ICC	Intraclass correlation coefficient
IIH	Idiopathic intracranial hypertension
IQR	Interquartile range
MD	Mean diffusivity
MRI	Magnetic resonance imaging
PLIC	Posterior limb of internal capsule
RD	Radial diffusivity
ROC	Receiver operating characteristic
ROI	Region of interest
SAS	Subarachnoid space
SD	Standard deviation
TBSS	Tract-based spatial statistics
